# Release of natural extracts from PVA and PVA-CMC hydrogel wound dressings: a power law swelling/delivery

**DOI:** 10.3389/fbioe.2024.1406336

**Published:** 2024-08-06

**Authors:** Renata Nunes Oliveira, Luiz Augusto da Cruz Meleiro, Brid Quilty, Garrett Brian McGuinness

**Affiliations:** ^1^ Chemical Engineering Department, Institute of Technology, Federal Rural University of Rio de Janeiro, Rio de Janeiro, Brazil; ^2^ School of Biotechnology, Dublin City University, Dublin, Ireland; ^3^ School of Mechanical and Manufacturing Engineering, Dublin City University, Dublin, Ireland

**Keywords:** Dragon’s blood, sage extract, PVA, hydrogel, wound dressing

## Abstract

**Introduction:**

PVA hydrogels present many characteristics of the ideal dressing, although without antimicrobial properties. The present work aims to study the physical, mechanical and release characteristics of hydrogel wound dressings loaded with either of two natural herbal products, sage extract and dragon's blood.

**Methods:**

Fourier Transform Infrared Spectroscopy (FTIR), Differential Scanning Calorimetry (DSC) and tensile mechanical testing were used to investigate the structure and properties of the gels. Swelling and degradation tests were conducted according to ISO 10993-9. Release characteristics were studied using UV Spectrophotometry.

**Results:**

PVA matrices incorporating sage extract or dragon's blood (DB) present hydrogen bonding between these components. PVA-CMC hydrogels containing sage present similar spectra to PVA-CMC alone, probably indicating low miscibility or interaction between the matrix and sage. The opposite is found for DB, which exhibits more pronounced interference with crystallinity than sage. DB and NaCMC negatively affect Young's modulus and failure strength. All samples appear to reach equilibrium swelling degree (ESD) in 24 h. The addition of DB and sage to PVA increases the gels' swelling capacity, indicating that the substances likely separate PVA chains. The inclusion of CMC contributes to high media uptake. The kinetics profile of media uptake for 4 days is described by a power-law model, which is correlated to the drug delivery mechanism.

**Discussion:**

A PVA-CMC gel incorporating 15% DB, the highest amount tested, shows the most favorable characteristics for flavonoid delivery, as well as flexibility and swelling capacity.

## 1 Introduction

Skin is a vast complex organ, responsible for protecting the body, as well as for regulation of homeostasis. The epidermis is the external layer of skin, which prevents the penetration of stressors, while the dermis is the second (internal) layer containing fat and is responsible for the elasticity of the skin (Cua et al., 1990). Older skin has limited repair capacity and a slow wound healing rate, which in turn facilitates the development of chronic wounds, especially if the patient already has a chronic condition (Takahashi et al., 2004; Xiao et al., 2022). Dry skin and itching may result in a wound, which allied to low vascularization and innervation, may delay healing (Bánvölgyi et al., 2022). A hydrogel is a hydrophilic polymeric gel layer, physical or chemically crosslinked, presenting structural integrity when hydrated. Hydrogels are typically non-adherent to the wound site, relieve pain, stimulate autolytic *debridement* and present several characteristics associated with ideal dressings (Brumberg et al., 2021).

Hydrogels that are biocompatible and absorb wound exudate while swelling in biological fluids stimulate the healing process, especially biodegradable hydrogels containing reactive oxygen species (Yu et al., 2022). Among hydrogels, *in-situ* chemically crosslinked hydrogels can bind effectively to living tissue, protecting wounds from exterior damage, as well as replicating the barrier properties, vapor transmission and other functions of skin) (Yang et al., 2016). On the other hand, physically crosslinked hydrogels are also interesting for wound healing and can be manufactured to reach properties that mimic skin. Double-network hydrogels have promise for wearable biosensors (monitoring biological fluids), drug delivery systems and scaffolds (Guo et al., 2023).

Traditional hydrogels protect the wound site from secondary infection by preventing permeation of microorganisms toward the wound. However, to treat infected wounds, antibacterial hydrogels are recommended. These gels kill bacteria and allow oxygen transport which stimulates healing (Liu et al., 2022). Infection is a main risk factor for elderly people suffering with wounds (Mattera et al., 2014). Many senior people live in long-term care facilities, places in which infection by methicillin resistant *Staphylococcus aureus* (MRSA) is potentially found (Augustine and Bonomo, 2011). Natural products such as honey have been used as a wound covering material, presenting faster and complete healing with low odour (Zeleníková and Vyhlídalová, 2019). In another example, infection caused by antibiotic resistant *S. aureus* and *Pseudomonas aeruginosa* (bacteria that colonize wounds) have been successfully treated by the synergistic effect of *Rumex abyssinicus* and *Discopodium penninervium* in a blend of essential oils (Gadisa and Usman, 2021). MRSA infection has also been successfully treated *in-vitro* by *Cinnamomum impressicostatum* stem-bark water extract (Buru et al., 2014).

*Salvia officinalis* (sage) hydroalcoholic extracts have been shown to present anti-inflammatory and antioxidant activities. Sage has been shown to regulate the expression of pro-inflammatory cytokines, growth factors, and antioxidant properties, stimulating wound healing (Farahpour et al., 2020). Wounds treated with 5% Sage extract presented high re-epithelialization, wound contraction and angiogenesis (Karimzadeh and Farahpour, 2017). In addition, *S. officinalis* essential oil (which contains cis-thujone, camphor, trans-thujone and 1,8-cineole) was incorporated into gelatin hydrogels and their *S. aureus* and *E. coli* inhibition halos were similar to silver nanoparticles ones (Gherman et al., 2018). Dragon’s blood is a reddish resin extracted directly from the *Croton lechleri* tree (exudated by the tree stem when it is cut) (Peres et al., 2023). *Croton Lechleri* (Dragon’s blood) is a resinous material with anti-inflammatory, antioxidant and healing properties, probably attributable to phenolic compounds and alkaloid taspine (Gupta et al., 2008; Namjoyan et al., 2016). In Dragon’s blood, gallic acid, syringic acid, epicatechin and catechin are the most prevalent active substances (Diedrich et al., 2021). Dressings of oxidized hyaluronic acid and carboxymethyl chitosan loaded with Dragon’s blood from China showed sustained release of Loureirin A and Loureirin B (active compounds), accelerating healing of burns *in-vivo* (Xu et al., 2022).

Among the many hydrogels available for the incorporation of natural antibacterial products, common choices are polyvinyl alcohol (PVA) and sodium carboxymethyl cellulose (denoted ‘CMC’ in this work). PVA is a non-toxic, hydrophilic, biocompatible material while CMC is highly hydrophilic and a low-cost material. PVA and CMC can be mixed to adjust the resultant hydrogels’ functional properties (Djumaev and Tashmukhamedova, 2020). As an example of the usage of these gels as delivery systems, the release and erosion characteristics of PVA-CMC hydrogels laden with Inebrin have been successfully studied (Djumaev and Tashmukhamedova, 2020), as well as the release effect of PVA-CMC gels incorporating Cu nanoparticles and their positive activity against *S. aureus* and *E. coli* (Ponco and Helmiyati, 2020). As part of a broader study to evaluate natural products selected based on published evidence of their use as topical wound treatments, Sage extract and dragon’s blood, along with Propolis from two sources and Pomegranate, were previously characterized for their composition and antioxidant activity (Oliveira et al., 2016). Dragon’s blood exhibited amongst the highest anti-oxidant activity, but the dragon’s blood resin and Sage extract were both found to be in the lower range for phenol and flavonoid content. This means that the physical, chemical, swelling and release characteristics of the proposed PVA and PVA-CMC hydrogels are likely to be of critical importance to their overall efficacy in wound healing. The contribution of a detailed study of PVA-based gels incorporating dragon’s blood in particular provides insight into a system on which little information has previously been available.

## 2 Materials and methods

PVA and PVA-CMC samples were prepared through the dissolution of 10% w/v polymers (from 8 to 10 g of Poly (vinyl alcohol) - PVA, Mw 85.000–124.000 and degree of hydrolysis 99+%, Sigma-Aldrich^®^, and from 2 to 0 g of sodium carboxymethyl cellulose—NaCMC, 250000 Da, Sigma-Aldrich^®^), using distilled water (from 100 to 85 mL) and herbals (from 15 to 0 mL), Table 1. The natural products used were Sage Tincture (Fushi Wellbeing, 25%), labelled as “S”; and dragons’ blood, (Amazon Therapeutics Laboratories, 100%), named “DB”. These have been characterised in a previous publication via FTIR in terms of their chemical bond composition, and presence of phenols and flavonoids (Oliveira et al., 2016). The polymers (total of 10%, Table 1) were dissolved in distilled water at 90°C under mechanical stirring. After dissolution, the solution was kept under stirring until room temperature was reached. Then, the herbals were added under stirring. The amount of DB or S in each sample was based on the amount of each herbal added to the polymers, rather than a post-preparation measurement. 20 mL of each solution was poured on Petri dishes (φ 150 mm), freeze-thawed overnight (at −16°C), and dried in room conditions.

**TABLE 1:** 2^3^ Factorial design with three factors: “Polymer”, “Type of drug” and “Amount of drug”. Each factor consisted of 2 levels, respectively: PVA and PVA-CMC; Dragon’s blood and Sage extract; and from 0 mL to 15 mL of each herbal.

RunOrder	CenterPt	Polymer	Natural product	Amount of drug (mL)	Sample name
1	0	PVA	Dragon’s blood	7,5	PVA-7.5DB
2	1	PVA	Sage	15	PVA-15S
3	1	PVA-CMC	Sage	0	PVACMC
4	0	PVA-CMC	Sage	7,5	PVACMC-7.5S
5	0	PVA	Sage	7,5	PVA-7.5S
6	1	PVA-CMC	Dragon’s blood	15	PVACMC-15DB
7	0	PVA-CMC	Dragon’s blood	7,5	PVACMC-7.5DB
8	1	PVA	Dragon’s blood	0	PVA

### 2.1 Physical properties

The Fourier-Transform Infrared Spectroscopy (FTIR) analysis of the samples was performed. The equipment used was Perkin Elmer Spectrum GX (DCU), 16 scans per samples in the region of (4,000–650) cm^−1^.

The samples were analysed via differential scanning calorimetry (DSC), equipment Perkin Elmer, DSC 8000 (DCU). 10 mg of each sample was submitted to heating rate of 10°C/min from room temperature to 250°C. To overcome the thermal history of the samples, the second heating cycle was used to obtain the gels’ properties: glass temperature (Tg) and melting temperature (Tm). The degree of crystallinity (Xc) was calculated according to Eq. (1). (Yang et al., 2018; Zhu et al., 2020).

$$\chi_{c}\left(\%\right)=100\left(\frac{∆H}{w\left({∆H}_{0}\right)}\right)=100\left(\frac{∆H}{138.6w}\right)$$ (1)

w is the weight fraction of PVA; ΔH = sample’s heat of melting; ΔH_0_ = heat of melting of a pure crystalline PVA sample (138.6 J/g) (Jiang et al., 2014; Oliveira et al., 2019).

At least 6 samples of each composition were cut in dog-bone shape from the swollen hydrogels. After 1 day of swelling in PBS, the samples were submitted to tensile tests at room temperature, (Zwick Z005 Tensile Test Machine), with sandpaper in contact with the sample surface. The tests were performed using a 500 N load cell at a crosshead rate of 10 mm/min until failure. The results of at least 6 samples were evaluated, excluding the samples that presented the lowest and the highest curves. The ultimate tensile strength values (σ) and the Secant modulus values (E) were calculated.

Further characterization was based on the results of at least triplicates. *In-vivo* analysis could elicit further impacts in a living system but is beyond the scope of the present study.

### 2.2 Swelling and release results

Fluid absorption studies were performed using Phosphate Buffered Saline - PBS, Sigma Aldrich (0.01 M phosphate buffer, 0.0027 M KCl, 0.137 M NaCl), which mimics the inorganic phase of body fluids. The swelling/degradation tests followed the standard ISO 10993-9, where samples of approximately 5 cm^2^, weight normalized, were placed in 5 mL of each media. The samples remained in the media at 37°C for 4 days, being weighed at regular time intervals (1 h, 2 h, 4 h, 1 day, and 4 days) to calculate the samples’ Swelling Degree (SD) using Eq. (2). After 4 days, the samples were dried and weighed to calculate the gel fraction (GF) and weight loss (WL) using Eqs 3, 4. In these equations, SD_D_ is the dried samples weight before the swelling tests, SD_D2_ is the dried samples weight after the test, and SD_S_ is the swollen samples weight.

$$SD\left(\%\right)=100\left(\frac{{SD}_{S}-{SD}_{D}}{{SD}_{D}}\right)$$ (2)

$$GF\left(\%\right)=100\left(\frac{{SD}_{D2}}{{SD}_{D}}\right)$$ (3)

$$WL\left(\%\right)=100\left(\frac{{SD}_{D}-{SD}_{D2}}{{SD}_{D}}\right)$$ (4)

The kinetics of drug delivery from hydrogels has been extensively studied. Since delivery can occur simultaneously to the swelling, modelling the swelling degree of samples may indirectly describe the gels’ drug delivery. (Setapa et al., 2020). The SD of the samples was modelled by the Korsmeyer-Peppas (Power law) model (Eq. (5)), where $\frac{M_{t}}{M_{\infty }}$ is the fraction of drug released, k is the kinetic constant, t is the release time and n is the diffusional exponent of drug release. (Shabbir et al., 2018).

$$\frac{M_{t}}{M_{\infty }}=kt^{n}$$ (5)

The concentration of flavonoids delivered by the gels was measured. 0.5 mL of the swelling media after 4 days (1 mg/mL) was mixed with 0.5 mL of 2% AlCl_3_ (Sigma-Aldrich) ethanolic solution in the dark (triplicates). To plot a standard curve, 0.5 mL of different solutions of quercetin (Sigma-Aldrich, range of 0 μg−125.00 µg) in ethanol was mixed with 0.5 mL of 2% AlCl_3_ ethanolic solution in the dark. After incubation for 45 min, the solutions were analysed in the UV-Vis spectrophotometer (VWR, UV-3100PC Spectrophotometer, DCU), wavelength of 415 nm (Oliveira R.N. et al., 2016).

### 2.3 Statistical analysis

All data that was measured at least in triplicate (maximum of 6 samples) was statistically evaluated. Data with replicas were evaluated in OriginPro 8^®^ by 1-way ANOVA-analysis and Tukey test, where the factor was “composition”, with 8 levels (the compositions listed in Table 1), value of significance: 95%. All data was used as ‘response’ in the factorial design, but only the data showing significance in the pareto plot were included in the present work.

## 3 Results and discussion

### 3.1 Physical properties

#### 3.1.1 Fourier-transform infrared spectroscopy—FTIR

**FIGURE 1:**
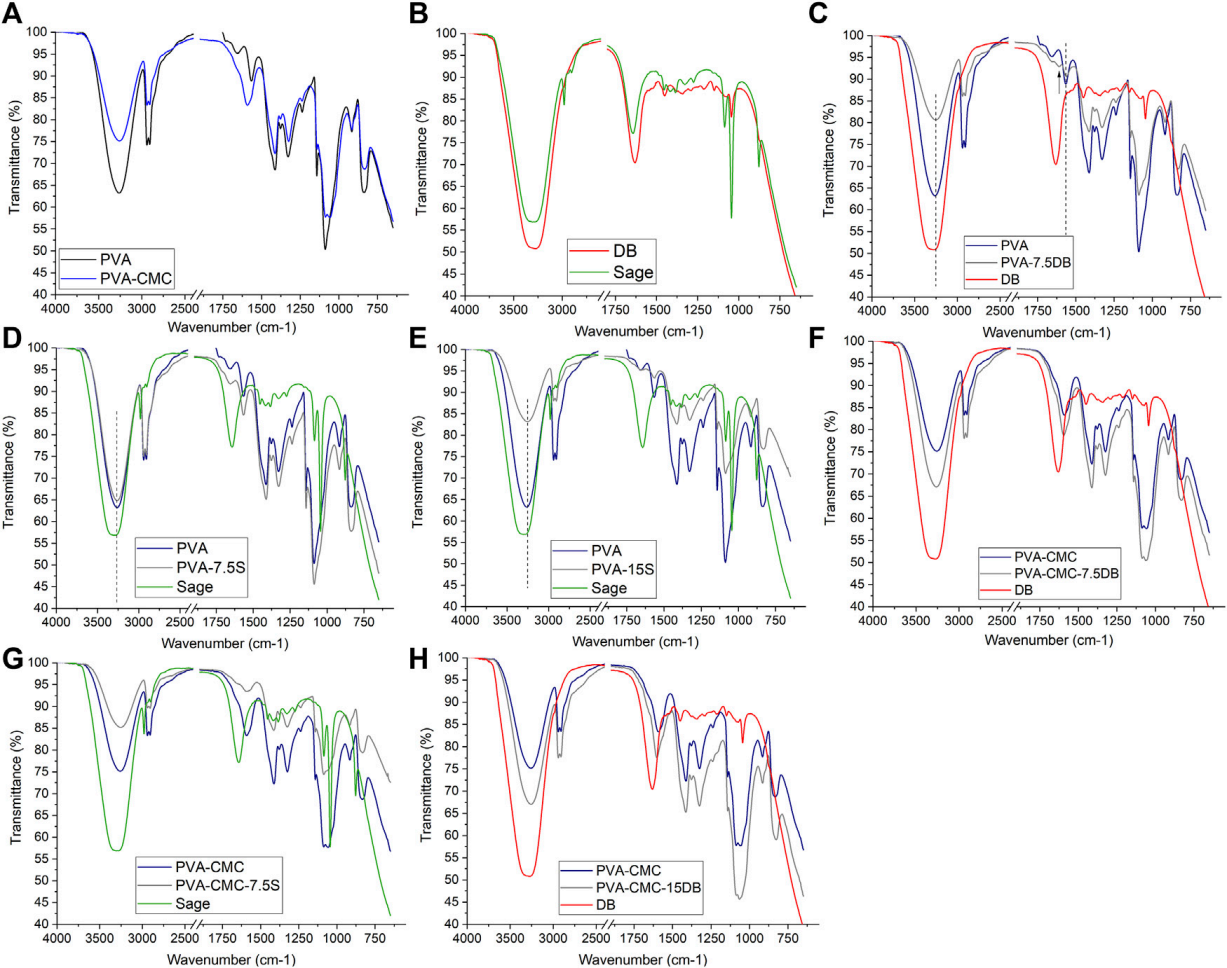
FTIR spectra of **(A)** the matrices, PVA and PVA-NaCMC; **(B)** the herbal extracts: sage, and dragon’s blood. FTIR spectra of the individual matrix and herbal, as well as the loaded samples: **(C)** PVA-7.5% DB sample; **(D)** PVA-7.5%S; **(E)** PVA-15%S; **(F)** PVA-CMC-7.5% DB; **(G)** PVA-CMC-15% DB; **(H)** PVA-CMC-7.5% S.

The polymeric matrices presented the characteristic bands of PVA and Na-CMC, Figure 1A and Table 2. Sage extract and dragon’s blood bands, Figure 1B and Table 1, revealed, besides the extracts/plants characteristic bands, an intense band at ∼3,300 cm^−1^, indicative of hydroxyl groups contribution of ethanol, since they are herbal extracts (Jadhav et al., 2017). In both spectra there is a band at approximately 1,080 cm^−1^, related to ethanol’s $\delta \left(C-O-C\right)$ vibration (Pangesti and Masruri, 2020).

**TABLE 2:** FTIR bands of the matrixes (PVA and PVA-CMC) and of the herbal extracts (dragon’s blood and sage).

Wavenumber (cm^−1^)	Vibration mode	References
PVA		
3,270	$\upsilon \left(-OH\right)$ of free alcohols	Choo et al. (2016); Dmitrenko et al. (2022); Siddaiah et al. (2018)
2,941	Alkyl’s $\upsilon \left(C-H\right)$	Reis et al. (2006), Mohamed et al. (2022)
2,908	$\upsilon \left(CH_{2}\right)$	Dmitrenko et al. (2022)
1,655; 1,567	$\upsilon \left(C=C\right)$	Choo et al. (2016), Siddaiah et al. (2018)
1,413	$\delta_{s}\left({CH}_{2}\right);\delta \left(O-H\right)$	Dmitrenko et al. (2022); Salazar (2018)
1,378	$\delta \left(C-H\right);\;\delta \left(O-H\right)$	Salazar (2018)
1,328	$CH;\;\delta \left(OH\right)$	Reis et al. (2006); Mohamed et al. (2022)
1,237	$\left(C-H\right)$	Zhang et al. (2020)
1,143	$\upsilon \left(C-O\right);\;\upsilon \left(C-C\right);crystallinity$	Reis et al. (2006), Salazar (2018)
1,088	$\upsilon \left(C-O\right)$	Choo et al. (2016); Salazar (2018); Siddaiah et al. (2018); Mohamed et al. (2022)
916	$\rho \left({CH}_{2}\right);\;\upsilon \left(C-C\right)$	Dmitrenko et al. (2022); Salazar (2018); Siddaiah et al. (2018)
840	$\upsilon \left(C-C\right)$ of planar zigzag carbon backbone	Choo et al. (2016); Dmitrenko et al. (2022)
PVA-CMC		
3,260	$\upsilon \left(-OH\right)$	Choo et al., 2016; Dmitrenko et al. (2022); Siddaiah et al. (2018); Sharratt et al. (2021)
2,940	PVA’s Alkyl’s $\upsilon \left(C-H\right);\text{ NaCMC}’s\text{ aliphatic }\upsilon \left(C-H\right)$	Reis et al. (2006); Kadry (2019); Mohamed et al. (2022)
2,908	PVA’s $\upsilon \left(CH_{2}\right);\text{ NaCMC}’s\;\upsilon \left(C-H\right)$	Dmitrenko et al. (2022); Devi et al. (2020)
1,591	NaCMC’s $\upsilon_{as}\left({COO}^{-}\right)$	Pettignano et al. (2019)
1,412	$\text{PVA}’s\;\delta_{s}\left({CH}_{2}\right);\;\delta \left(O-H\right);\text{ NaCMC}’s\;\upsilon_{as}\left({COO}^{-}\right)$	Dmitrenko et al. (2022), Salazar (2018); Kadry (2019)
1,378	$\text{PVA}’s\;\delta \left(C-H\right);\;\delta \left(O-H\right)$	Salazar (2018)
1,325	$\delta \left(OH\right)$	Reis et al. (2006); Devi et al. (2020); Mohamed et al. (2022)
1,238	$\text{PVA}’s\;\left(C-H\right)$	Zhang et al. (2020)
1,142	$\text{PVA}’s\;\upsilon \left(C-O\right);\;\upsilon \left(C-C\right);crystallinity$	Reis et al., 2006; Salazar, 2018
1,086	$\text{PVA}’s\;\upsilon \left(C-O\right);NaCMC^{\prime }s\;\upsilon \left(C-O-C\right)$	Choo et al. (2016); Salazar (2018); Siddaiah et al. (2018); Kadry (2019); Mohamed et al. (2019)
1,060	NaCMC’s $\upsilon \left(-C-O\right)$ of polysaccharide skeleton	Devi et al. (2020)
916	$\text{PVA}’s\;\rho \left({CH}_{2}\right);\;\upsilon \left(C-C\right)$	Dmitrenko et al. (2022); Salazar (2018); Siddaiah et al. (2018)
834	$\text{PVA}’s\;\upsilon \left(C-C\right)$ of planar zigzag carbon backbone	Choo et al. (2016); Dmitrenko et al. (2022)
Dragon’s Blood resin (DB)		
3,295	$\upsilon \left(-OH\right)$	Jadhav et al. (2017)
1,631	$\upsilon \left(C=C\right)$ of aromatics	Koperska et al. (2011); Silva and Rodriguez (2019)
1,451	Angular deformation of ( $\left.C-O\right)$ bond in $COOH;\;\upsilon \left(C=C\right)$ of aromatics	Koperska et al. (2011); Silva and Rodriguez (2019)
1,340	$\left(C-C\right)$	Koperska et al. (2011)
1,297	Angular deformation of $\left(C-O\right)$ bond in alcohols and phenols	Silva and Rodriguez (2019)
1,214	( $\left.C-O\right)$ of $COOH;\;\upsilon \left(O-{CH}_{3}\right)$ ; Angular deformation of (C-O) bond in alcohols and phenols	Koperska et al. (2011); Salguero and Pilaquinga (2011)
1,149	Angular deformation of $\left(C-O\right)$ bond in alcohols and phenols	Silva and Rodriguez (2019)
1,073		
1,045		
Sage Extract		
3,304	$phenolic\;\upsilon \left(-OH\right)$	Salević et al. (2022)
2,980	$\upsilon \left(C-H\right)$	Galvin-King et al. (2019)
2,904	$phenolic\;\left(-OH\right);\text{ aliphatic }\upsilon \left(C–H\right);\;{CH}_{3},{CH}_{2}\;\left(C_{sp}^{3},C_{sp}^{2}\right)$	Mot et al. (2022); Salević et al. (2022)
1,643	$carbonyl\;group^{\prime }s\;\upsilon \left(C=O\right);\upsilon \left(C=C\right)\;of\left(>C={CH}_{2}\right),\left(-\text{CH}=\text{CH}-\right),\left(-\text{CH}=C<\right)$	Mot et al. (2022)
1,454	$\left(C=C\right),\left(C=N\right),\left(C=O\right);\;\delta_{s,as}\left(C-H\right)\text{ of }{\text{CH}}_{2}\text{ and }{\text{CH}}_{3}\text{ groups};\;\delta_{in\;plane}\left(C-H\right);\;\upsilon_{s,as}\left(C-O\right);$ $\delta_{in\;plane}\left(O-H\right);\;\delta_{s}\left({CH}_{3},CO\right);\;\upsilon_{s,as}\left(C-O-C\right)$	Galvin-King et al. (2019); Mot et al. (2022)
1,418	$\upsilon ,\delta \left(\text{aromatic ring}\right)$	Salević et al. (2022)
1,385	$\left(C=C\right),\left(C=N\right),\left(C=O\right);\;\delta_{s,as}\left(C-H\right)\text{ of }{\text{CH}}_{2}\text{ and }{\text{CH}}_{3}\text{ groups};\;\delta_{in\;plane}\left(C-H\right);\;\upsilon_{s,as}\left(C-O\right);\;\delta_{in\;plane}\left(O-H\right);\;\delta_{s}\left({CH}_{3},CO\right);\;\upsilon_{s,as}\left(C-O-C\right)$	Galvin-King et al. (2019); Mot et al. (2022)
1,328		
1,275	$\delta \left(C-H\right);\;\delta \left(O-H\right)$	Salević et al. (2022)
1,086	$\delta_{s,as}\left(C-H\right)\text{ of }{\text{CH}}_{2}\text{ and }{\text{CH}}_{3}\text{ groups};\;\delta_{in\;plane}\left(C-H\right);$ $\upsilon_{s,as}\left(C-O\right);\;\delta_{in\;plane}\left(O-H\right);\;\delta_{s}\left({CH}_{3},CO\right);\;\upsilon_{s,as}\left(C-O-C\right)$	Mot et al. (2022)
1,044		
877	$\omega_{out-of-plane}\left({CH}_{2},CH\right);\;\delta_{out-of-plane}\left(O-H\right)$	Mot et al. (2022)

All samples presented the characteristic FTIR bands of the matrix and of the herbals. The differences encountered are highlighted as follows, firstly the PVA matrix samples and then, PVA-matrix samples. The PVA matrices loaded with the herbals (sage or DB), Figure 1, presented displacement of the PVA bands at 3,270 cm^−1^ ( $\upsilon \left(-OH\right)$ ) (Choo et al., 2016; Dmitrenko et al., 2022; Siddaiah et al., 2018) and at 840 cm^−1^ ( $\upsilon \left(C-C\right)$ ) (Choo et al., 2016; Dmitrenko et al., 2022) toward lower wavenumbers (indicated by dash lines in Figures 1C–E). Since these bands at lower wavenumber were not identified in the isolated materials (PVA, sage or dragon’s blood), they might be related to physical interactions between components. Since both herbals and matrices present -OH groups, hydrogen bonding may have occurred (Fathi et al., 2011). The $\upsilon \left(C-C\right)$ displacement could be attributed to Wan der Waals interactions between the matrix and the herbal extract. PVA-7.5DB sample presented displacement of a band from 1,567 cm^−1^ ( $\upsilon \left(C=C\right)$ ) (Choo et al., 2016; Siddaiah et al., 2018) to 1,560 cm^−1^, as well as the presence of a band at 1,608 cm^−1^ (indicated by an arrow in Figure 1C), besides all bands of PVA and DB. This could be related to oxidative process (Abdel Bary et al., 2018), as well as to specific chemical interaction between PVA and DB (Akhlaq et al., 2021).

The PVA-CMC-7.5DB and PVA-CMC-15DB (named in Table 1) presented patterns similar to PVA-CMC. The slight changes were the overlap of the DB bands with the PVA-CMC ones (Figures 1F,G) (Neres Santos et al., 2019). The similarity to the PVA-CMC spectrum probably indicates low miscibility or interaction between the matrix and sage extract (da Silva Cabral and Jose Felix Carvalho, 2019). There was physical interaction between PVA and the herbals, and an oxidative process was identified in PVA-7.5 DB sample. PVA-CMC samples presented mostly PVA-CMC bands and low influence of the herbal bands.

#### 3.1.2 Differential scanning calorimetry - DSC

**FIGURE 2:**
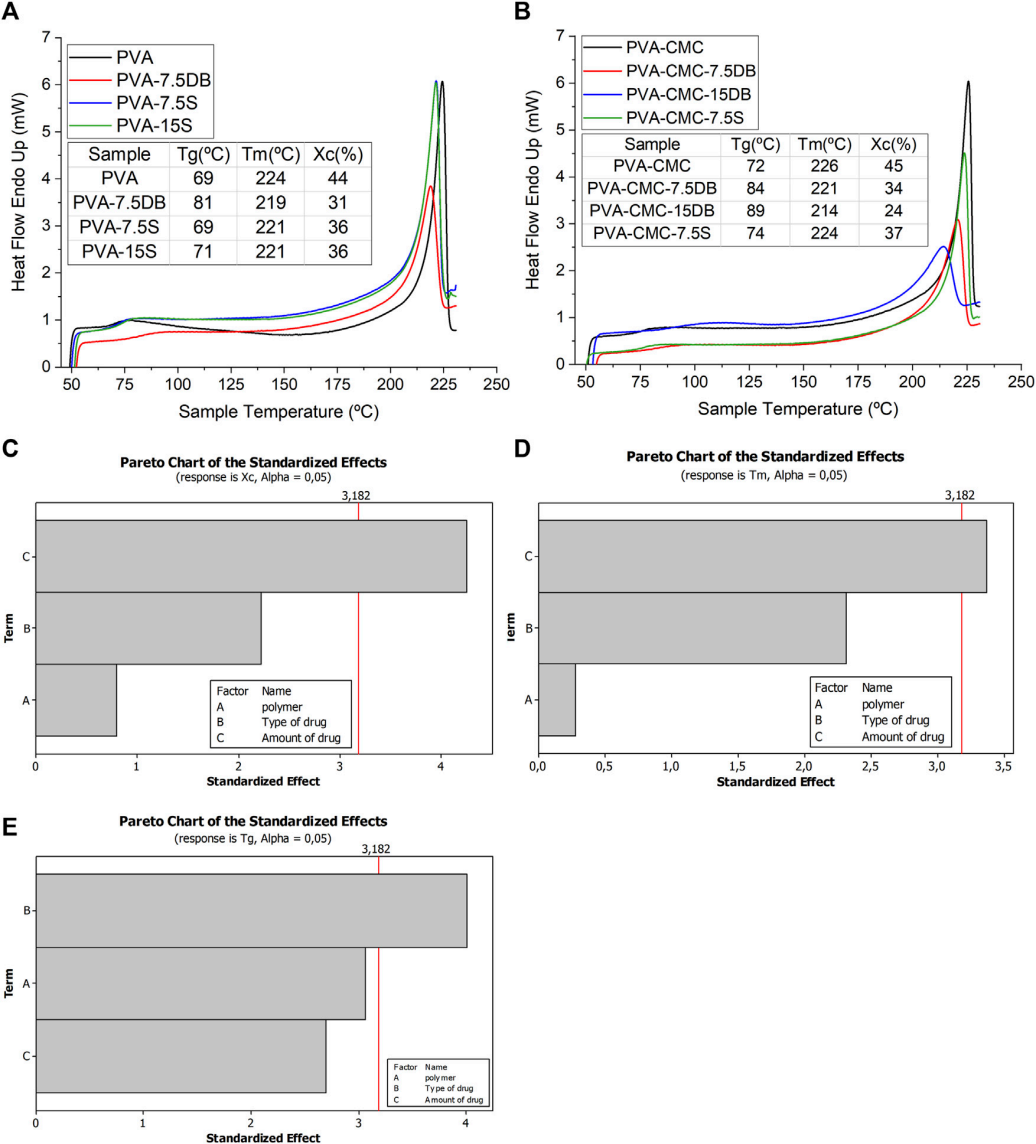
DSC curves of **(A)** PVA samples loaded with herbals; **(B)** PVA-CMC samples loaded with herbals. Pareto plots of **(C)** Xc, **(D)** Tm, **(E)** Tg.

The polymeric samples loaded with natural products presented higher Tg and lower Tm and Xc compared to the polymeric matrices themselves (PVA and PVA-CMC samples), Figure 2. By adopting Tg, Tm and Xc data as factorial design responses, it was observed that the amount of extract was a significant factor for Xc and Tm, while the type of extract was the determinant parameter for the Tg results, Figures 2C–E. The addition of Dragon’s blood resulted in higher Tg than for sage extract. High Tg in the presence of the extracts means that the amorphous polymeric chains need more heat for chains to move. High Tg would be due to hydrogen bonding formation between chains, as the FTIR spectra indicated by changes in the spectra (Campos et al., 2013). Low Tm and Xc represent interference of the herbals with the ability of chains to pack into crystallites, resulting in fewer imperfect crystallites being formed (Peng et al., 2015). There is a lower transition temperature from amorphous to crystalline (Siddaiah et al., 2018). In addition, the low Xc indicates that DB interferes with contact between PVA chains and that there is compatibility or miscibility between PVA and DB components compared to sage, in agreement with the FTIR results (Restrepo et al., 2018). It is clearly observed that DB interferes with the samples’ crystallinity (lowering it) more pronouncedly than sage. The samples containing sage present high crystallinity, where the Tm is slightly shifted to low temperatures, Figures 2F–H. This could be attributed to the effect of the herbals’ molecules being positioned between the polymer chains, weakening the connections between them (Campos et al., 2013).

Amorphous and crystalline gels result in different rates of degradation (weight loss - WL) and drug delivery rate. More crystalline gels usually present slow WL and drug delivery, with samples presenting the highest Xc and Tm being the slowest. By that measure, the best composition would be PVA-CMC without any herbal constituent. However, since the goal of this work was to slowly delivery phenols and flavonoids to the wound site, it is observed that PVA-CMC loaded with sage presented the highest Xc and Tm among the studied samples. These gels also present relatively low Tg, indicating facilitation of the movement of chains in the matrix, which would make the membranes more conformable. Samples with low Tg, Tm and Xc would be soft and comfortable to user.

#### 3.1.3 Mechanical properties

**FIGURE 3:**
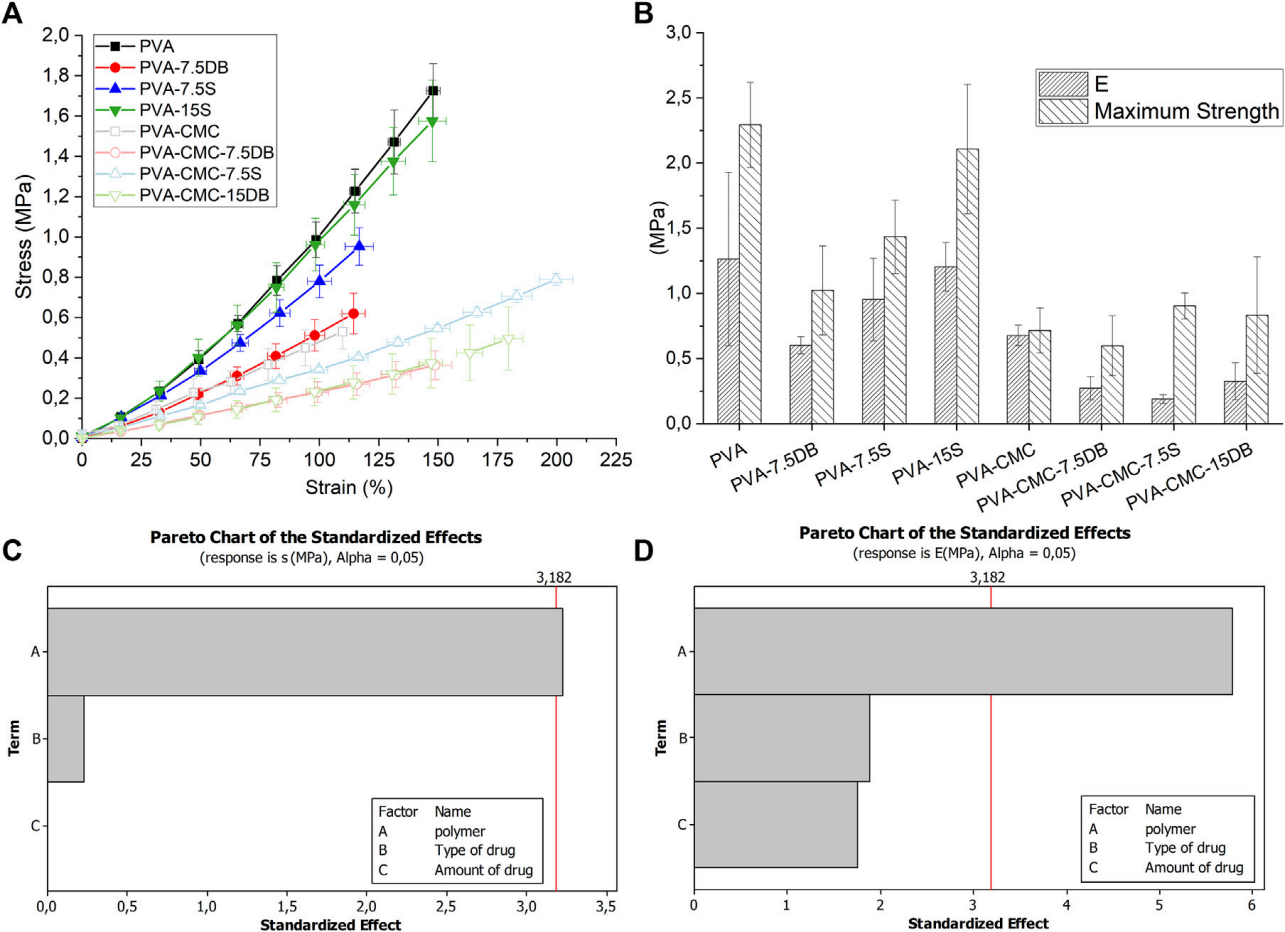
Samples **(A)** mechanical results; **(B)** Young modulus, “E”, and failure strength, “s”; samples’ Pareto plots of **(C)** failure strength and **(D)** Young’s modulus.

The mechanical properties of swollen samples were evaluated by ANOVA−1 way analysis (factor “composition”, 8 levels) as well as by the MINITAB software regarding the factorial design responses (average results of Young’s modulus, “E”, and failure strength, “s”), Figure 3. The type of matrix (PVA or PVA-CMC) is the significant parameter for the mechanical properties studied (Figure 3, Pareto plots).

PVA-CMC presents a low Young’s modulus and failure strength compared to PVA samples. Since NaCMC mechanical properties are considered poor compared to the PVA ones, this trend was expected (Tarawneh et al., 2021; Cyriac et al., 2022). Sodium carboxymethyl cellulose is obtained through carboxymethylation reaction, where low weight molecules can be extracted from the cellulose fibres. These oligomers may act as plasticizers (“small” molecules between polymeric chains, separating them), resulting in poor mechanical properties (Siqueira et al., 2015).

DB and NaCMC negatively affect the Young’s modulus (p< 0.05), which is in line with the Tm and Xc results from DSC. Since Young’s modulus is associated with the degree of bonding strength (Zeinalipour-Yazdi and Christofides, 2009), NaCMC is believed to interfere with PVA chain packing. NaCMC also diminishes failure strength (p< 0.05). The more extensible gels are, the more comfortable they may be to the patient. In the present work, the stiffness of all gels were similar or higher than 0.02–0.05 MPa, which is the reported skin stiffness of patients older than 30 years-old (Joodaki and Panzer, 2018). The lower stiffness gels were considered the most promising ones, specifically PVA-CMC gels loaded with 15% S or 15% DB.

#### 3.1.4 Swelling and release results

**FIGURE 4:**
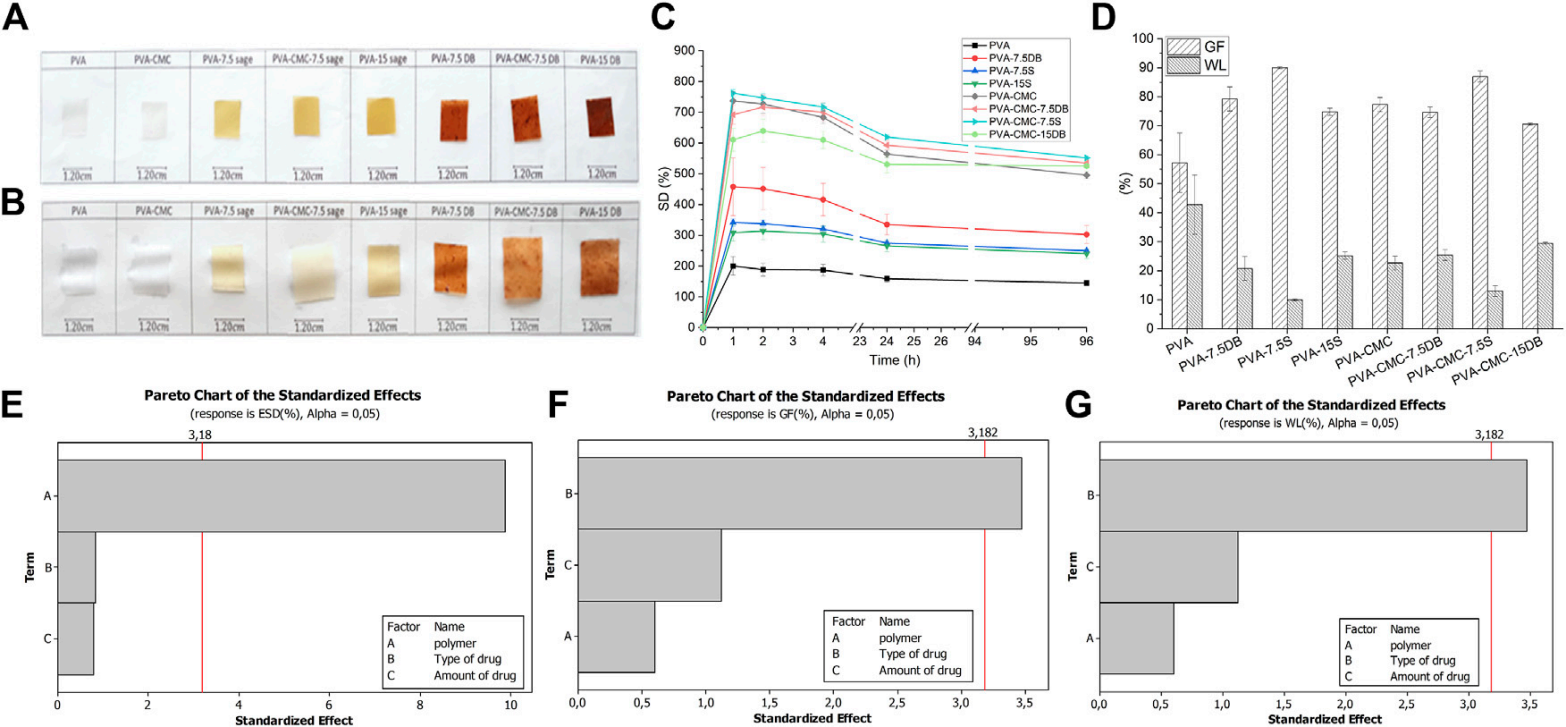
**(A)** original samples; **(B)** swollen (for 2 h) samples. Samples’ **(C)** swelling degree; **(D)** gel fraction and weight loss; **(E)** Pareto chart of ESD average value; **(F)** Pareto chart of gel fraction; **(G)** Pareto chart of weight loss.

The samples swelling capacities are shown in Figure 4. After 2 h of swelling, the size gain is visible (Figures 4A,B). The samples seemed to reach the equilibrium swelling degree (ESD) in 24 h, Figure 4C. ESD is determined by the equilibrium between the elastic forces of the network and the polymer relaxation (Catoira et al., 2019). The type of matrix (factor “polymer”) determines the significant differences between the samples swelling degree (p< 0.05), where samples with CMC present higher swelling capacity, Figure 4 (c, e, h). The high water uptake of gels containing NaCMC is expected, since NaCMC hydrogels are considered superabsorbent, influenced by the high free volume of the network allied with the high hydrophilicity of NaCMC (Basu et al., 2018; Soares et al., 2022). CMC contributes to high media uptake, since it is a highly hydrophilic polysaccharide, diminishing the overall Tm and Xc (Karoyo and Wilson, 2021). Gels/films with high ESD would easily deliver the herbals to the wound site and keep it moisturized (Firlar et al., 2022). In these gels, more water molecules enter the gels’ pores and can change places with the herbal substances, thereby delivering them to the wound site. PVA-CMC gels loaded with 15% S or 15% DB could be the ones with the most potential.

Gel fraction and weight loss present opposite trends since they represent the opposite side of the same effect. Weight loss measures the samples’ ability to hydrolytically degrade in media (Zhan and Chu, 2002), while gel fraction is an indirect measurement of physical crosslinking (related to effective amorphous entanglements and to PVA crystallites) (Rajawasam et al., 2024). The type of active substance interfered with both gel fraction and weight loss: sage extract resulted in higher gel fraction and lower weight loss compared to dragon’s blood, Figure 4. Dragon’s blood presented poor water solubility, and if it remained between polymeric chains (Wang et al., 2023), the contact between chains could then be lower, leading to low gel fraction and high weight loss. Although sage extract (hydroalcoholic extract) may also be located between PVA chains, it is a polar extract (Somayeh Samaei et al., 2021), together with water, which facilitates the molecules to exchange places. CMC contributes to high media uptake, since it is a highly hydrophilic polysaccharide, diminishing the overall Tm and Xc (Karoyo and Wilson, 2021). Gels/films with high ESD would easily deliver the herbals to the wound site and keep it moisturized. (Firlar et al., 2022). In these gels, more water molecules enter the gels’ pores and can change places with the herbal substances, thereby delivering them to the wound site. PVA-CMC gels loaded with 15% S or 15% DB could be the ones with the most potential.

**FIGURE 5:**
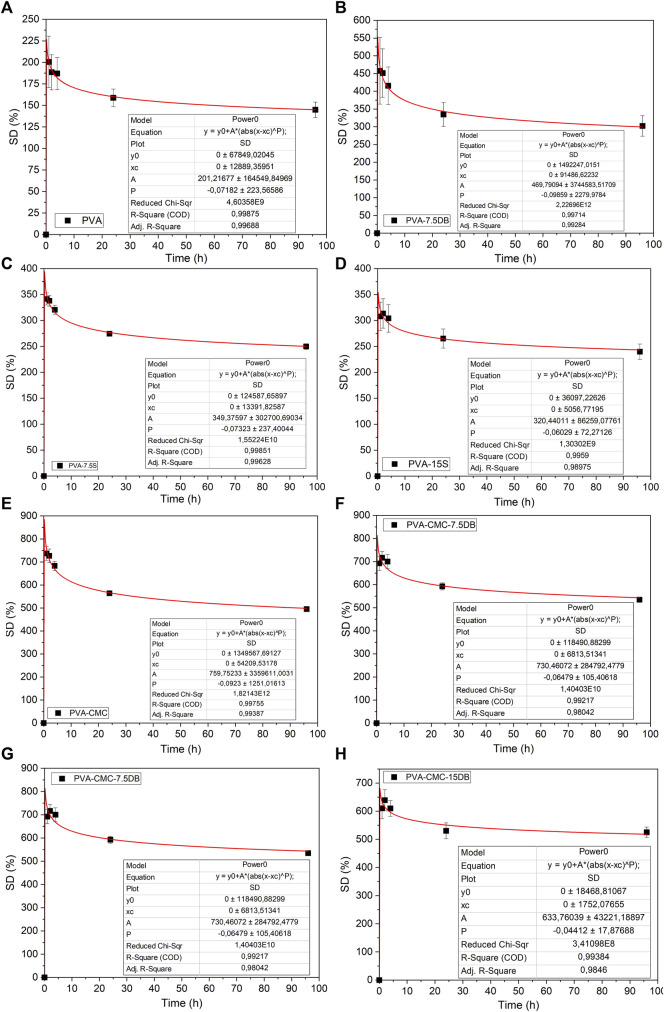
Samples **(A–H)** swelling degree modelling by the Power law.

The swelling degree was modelled by the Ritger-Peppas equation (Ritger and Peppas, 1987) (p< 0.05), Eq. (5) and Figure 5. Samples’ swelling ability was modelled and it is noted that a power-law defines the samples water uptake profile $\left(R^{2}\geq 0.99\right)$ . The Peppas equation (Power-law model) is used for drug delivery mechanisms, mainly applied for the first 60% of release, where a Fickian mechanism is proposed (Mircioiu et al., 2019). Although the Peppas model usually describes initial drug release (no more than 60% of release), Korsmeyer-Peppas has also been used to model sustained release (Ge et al., 2022). The Fickian mechanism would be a dose-dependent model, usually occurring below the ESD, when the media penetrates due to the matrix chains mobility and water forces (Ozcan and Didem Saloglu, 2015). When modelling drug release with the Korsmeyer-Peppas equation, the diffusional coefficient “P”<0.5 means that drug release is fast, since it is determined by the water entrance. The interactions between drug and matrix are weak (Frachini et al., 2023), and the hydrogels’ swelling follows a Fickian diffusion model (Saidi et al., 2020). Diffusion modelling is usually associated with steric interactions when the hydrogel pores are larger than the active molecules (pores would work as physical reservoirs for the active substance) (Axpe et al., 2019).

##### 3.1.4.1 Flavonoid release

**FIGURE 6:**
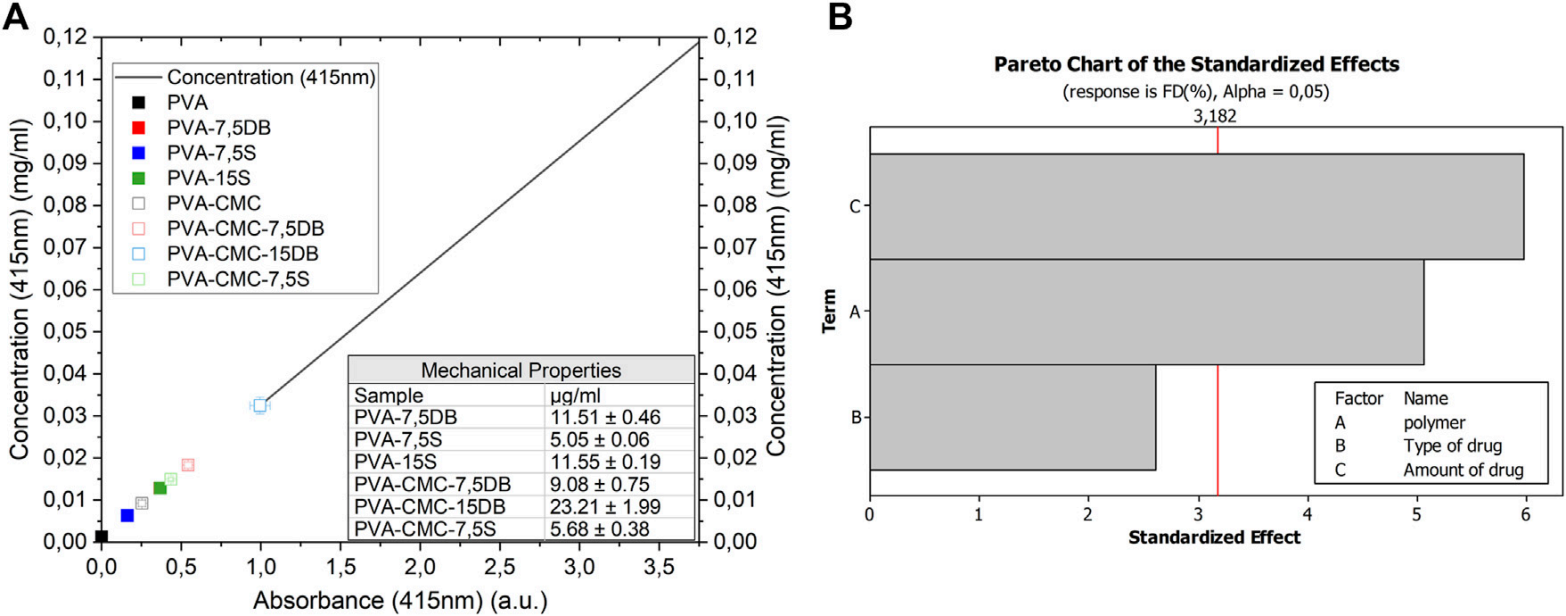
Samples’ **(A)** Flavonoids delivery; **(B)** pareto chart of effects on the amount of Flavonoids delivery.

Since PVA and PVA-CMC systems have not previously presented the desired antimicrobial activity (Neres Santos et al., 2019), Dragon’s blood and sage extract, containing flavonoids and other active components to control infection, were added. To quantify flavonoid delivery, a standard curve based on quercetin was plotted and the samples’ swelling media was evaluated, Figure 6. It can be observed that the PVA-CMC sample interfered with the light transmittance at 415 nm. This is probably due to products of the matrix degradation in the media. To avoid this interference, the values of PVA and PVA-CMC samples were diminished for their respective loaded samples for further analysis. There is a significant difference (*p* = 4,49 E^-14^) on the concentration of flavonoids delivered, where flavonoid delivery was mainly determined by the type of herbal used, as well as the amount. Since the flavonoids molecules were much smaller than the gels pore size, their diffusion would be quick. If not, it would indicate that the released substances sizes match the samples’ pore size, resulting in slow diffusion (Vigata et al., 2020). As expected, high amounts of herbal in the sample led to a high concentration of flavonoids delivered. Flavonoids are part of the polyphenol substances that usually present limited availability in hydrophilic media due to their lipophilic nature (Costa et al., 2021). Usually, flavonoid activity in wound healing is a synergistic effect of a variety of anti-inflammatory compounds (Carmignan et al., 2020). The addition of DB resulted in almost double the flavonoid delivery compared to sage, and DB is known for accelerating diabetic foot ulcer healing (Rempel et al., 2020). PVA-CMC-15DB would be the preferred sample for flavonoids delivery.

The activity of DB was highlighted in a clinical trial (random, double-blinded study, comprising 60 patients from 14 to 65 years old) when a topic cream loaded with DB significantly diminished the healing times of wounded patients (*p* = 0,0001) (Namjoyan et al., 2016). DB, due to substances present in the dragon’s blood, e.g., proanthocyanidins, stimulates wound contraction, formation of new collagen, crust formation and regeneration of the epithelium (Pieters et al., 1995). Regarding Sage, creams with sage extracts led to hydroxyproline production, positively influenced macrophage and fibroblasts distribution, stimulating collagen production and accelerating wound healing (Marques et al., 2023). Characterization of the overall biological response elicited by the hydrogels in the present study, which will be dependent on their composition and relevant to their tissue healing function, is an important future step in this work (Li et al., 2023; Luo et al., 2023).

## 4 Conclusion

PVA matrices loaded with herbal extracts (sage or DB) presented hydrogen bonding between components. DB interferes with PVA chain contact and there is greater compatibility/miscibility between PVA and DB components compared to sage. It is clearly observed that DB interferes with crystallinity more pronouncedly than sage. DB and NaCMC negatively affect Young’s modulus and failure strength. The addition of DB and sage to PVA increased swelling capacity, indicating that the herbal substances remained between PVA chains. PVA-CMC gels loaded with 15% S or 15% DB appear to have better potential regarding high ESD. The kinetics profile of media uptake for 4 days was described by Peppas-Ritger model, which is correlated to the drug delivery mechanism. PVA-CMC-15DB emerged as the most promising system for flavonoid delivery, ESD, and antioxidant activity. This material should also moisturize the wound site while it releases antimicrobial substances. Further, longer term studies are required to assess the antimicrobial effect in practice, in order to confirm potential to promote the overall healing process.

## Data Availability

The original contributions presented in the study are included in the article/supplementary material, further inquiries can be directed to the corresponding author.
